# Supplementary material for: TLR2 Signaling Pathway Combats Streptococcus uberis Infection by Inducing Mitochondrial Reactive Oxygen Species Production
Source: Cells. 2020 Feb 21;9(2):494. doi: 10.3390/cells9020494 (PMC7072855; doi:10.3390/cells9020494)
Supplement: Supplementary file 1 [file cells-09-00494-s001.pdf]

Table S1. Oligonucleotide sequences used for qPCR.

Gene	Primers sequence (5′-3′)	Orientation
**GAPDH**	GTTCAACTATTGGTGCTGG	Forward
	TCATTAGGTCCCGTTTGT	Reverse
**TNF-α**	CATCTTCTCAAAATTCGAGTGACAA	Forward
	TGGGAGTAGACAAGGTAGAACCC	Reverse
**IL-1β**	AACCTGCTGGTGTGTGACGTTC	Forward
	CAGCACGAGGCTTTTTTGTTGT	Reverse
**IL-6**	TGGAGTCACAGAAGGAGTGGCTAAG	Forward
	TCTGACCACAGTGAGGAATGTCCAC	Reverse
**TLR2**	TGCAAGTACGAACTGGACTTCT	Forward
	CCAGGTAGGTCTTGGTGTTCATT	Reverse
**TLR4**	TAGCCATTGCTGCCAACATCAT	Forward
	AAGATACACCAACGGCTCTGAA	Reverse

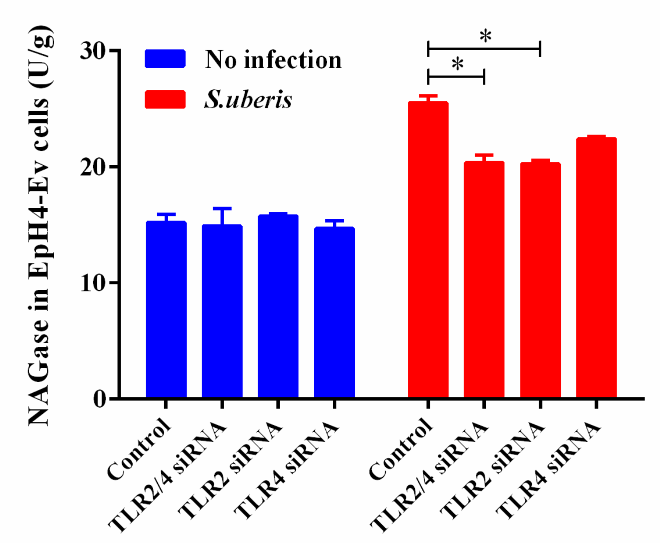

Figure S1 TLR2/4 mediate the NAGase activity after challenge with *S. uberis* in the supernatant of MECs. Experiments were repeated three times and data were presented as the means ± SEM (n = 3). *(*P*< 0.05) = significantly different between the indicated groups.

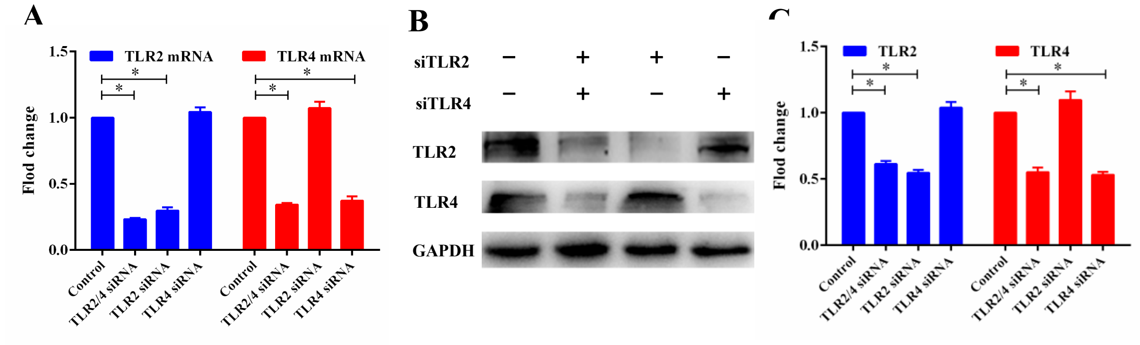

Figure S2 TLR2/4 mediate inflammatory response after challenge with *S. uberis* in MECs. MECs were transfected with 50nM siTLR2 or/and siTLR4 for 72 h. Then cells were infected with *S. uberis* in mid-exponential phase at a multiplicity of infection (MOI) of 10 for 3 h at 37 °C. The modulating effects of siRNAs were confirmed by RT-qPCR (A) and western blotting (B, C). Experiments were repeated three times and data were presented as the means ± SEM (n = 3). *(*P*< 0.05) = significantly different between the indicated groups.

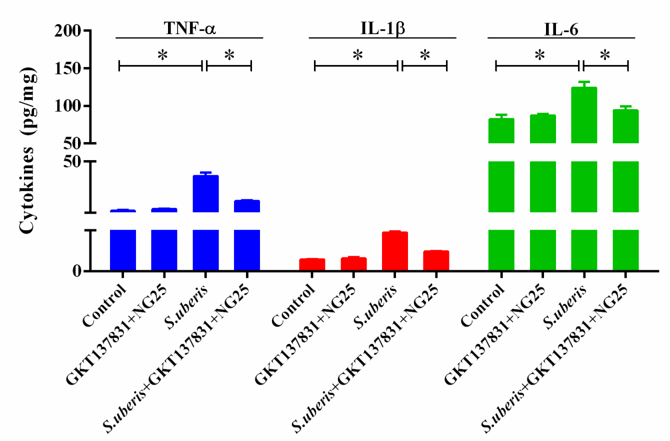

Figure S3 Limiting mROS reduces the inflammation factors after challenge with *S. uberis* in the supernatant of MECs. Experiments were repeated three times and data were presented as the means ± SEM (n = 3). *(*P*< 0.05) = significantly different between the indicated groups.

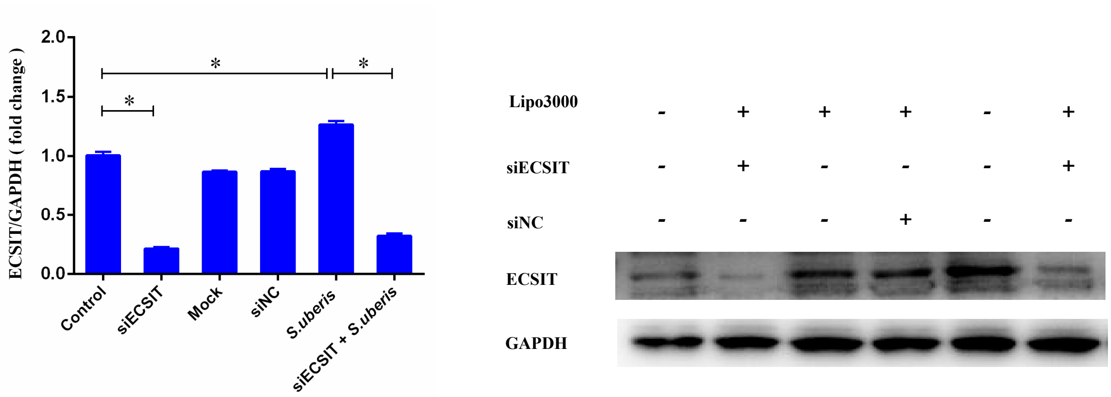

Figure S4 The protein expression of ECSIT were determined by Western blot after using siECSIT in MECs. Experiments were repeated three times and data were presented as the means ± SEM (n = 3). *($P < 0.05$) = significantly different between the indicated groups.
